# Taro raphide‐associated proteins: Allergens and crystal growth

**DOI:** 10.1002/pld3.443

**Published:** 2022-09-02

**Authors:** Robert E. Paull, Dessireé Zerpa‐Catanho, Nancy J. Chen, Gail Uruu, Ching Man Jennifer Wai, Michael Kantar

**Affiliations:** ^1^ Tropical Plant and Soil Sciences University of Hawaii at Manoa Honolulu HI USA; ^2^ Department of Plant Biology University of Illinois at Urbana‐Champaign Urbana IL USA; ^3^ Department of Horticulture Michigan State University East Lansing MI USA

**Keywords:** actin, biomineralization, calcium oxalate, crystalloplastids, profilin, transvacuolar strands

## Abstract

Calcium oxalate raphide crystals are found in bundles in intravacuolar membrane chambers of specialized idioblasts cells of most plant families. Aroid raphides are proposed to cause acridity in crops such as taro ( *Colocasia esculenta* (L.) Schott). Acridity is irritation that causes itchiness and pain when raw/insufficiently cooked tissues are eaten. Since raphides do not always cause acridity and since acridity can be inactivated by cooking and/or protease treatment, it is possible that a toxin or allergen‐like compound is associated with the crystals. Using two‐dimensional (2D) gel electrophoresis and mass spectrometry (MS) peptide sequencing of selected peptides from purified raphides and taro apex transcriptome sequencing, we showed the presence on the raphides of peptides normally associated with mitochrondria (ATP synthase), chloroplasts (chaperonin ~60 kDa), cytoplasm (actin, profilin), and vacuole (V‐type ATPase) that indicates a multistage biocrystallation process ending with possible invagination of the tonoplast and addition of mucilage that may be derived from the Golgi. Actin might play a crucial role in the generation of the needle‐like raphides. One of the five raphide profilins genes was highly expressed in the apex and had a 17‐amino acid insert that significantly increased that profilin's antigenic epitope peak. A second profilin had a 2‐amino acid insert and also had a greater B‐cell epitope prediction. Taro profilins showed 83% to 92% similarity to known characterized profilins. Further, commercial allergen test strips for hazelnuts, where profilin is a secondary allergen, have potential for screening in a taro germplasm to reduce acridity and during food processing to avoid overcooking.

## Highlights

All edible Aroids cause allergic type responses (acridity) thought to be caused by the sharp calcium oxalate raphide crystals. We have shown that raphide‐associated proteins could guide biomineralization, and the profilins found are potential allergens.

## 1 INTRODUCTION

Taro, *Colocasia esculenta*, (L.) Schott., and to a lesser extent Xanthosoma spp. are a staple crop estimated to be consumed by about half a billion people (Lebot, 2009; Talwana et al., 2009). Taro is the fifth most important root crop worldwide (Lebot, 2009) and when handled can cause skin edema or when eaten without cooking can cause swelling of lips, mouth and throat, itchiness, and pain. This irritation is referred to as acridity. The cause of acridity is unknown (Bradbury & Holloway, 1988; Bradbury & Nixon, 1998; Paull et al., 1999; Tang & Sakai, 1983). No quantitative assay is available except for a subjective estimation of acridity after eating or rubbing on the forearm. The most common explanation is that the needle‐like calcium oxalate raphides found in idioblasts that cause the acridity by mechanical means (Bown, 2000; Cody & Cody, 1987; Foster, 1956; Matthews, 2004; Payne et al., 1941; Pedler & Warden, 1888; Tanaka et al., 2003c; Watson et al., 2005). The calcium oxalate content in taro is genetically controlled, and large variation exists across taro varieties (Lewu et al., 2009; TANSAO, 2002). The density of raphides is higher in taro leaves and lower in the corm (Tanaka et al., 2003a, 2003b) and highest in young fully furled leaves and is less in the mature unfurled leaves (Sunell & Healey, 1985). Taro grown in the shade and under wet conditions has fewer raphide bundles (Tanaka et al., 2003b). However, a lower number of raphide bundles does not mean a species or tissue is less acrid. Idioblast density in taro is a polygenic trait with a heritability ~.5 (Tanaka et al., 2003b), though no acridity heritability data are available, in the absence of an assay.

Raphides, styloids, and druses are various types of calcium oxalate crystals found in more than 200 families in both dicotyledonous and monocotyledonous plants (Gallaher, 1975). The distinctive shapes and sizes of these crystals have some taxonomic value (Franceschi & Horner, 1980; Nakata & McConn, 2003; Prychid & Rudall, 1999; Raman et al., 2014; Webb, 1999). The idioblasts with their extensive endoplasmic reticulum, enlarged nucleus and nucleolus, and depleted stores of lipid and starch are found in developing parenchyma tissue of most plant organs (Horner & Wagner, 1995; Kausch & Horner, 1983; Pennisi et al., 2001; Prychid & Rudall, 1999). In Lemna, the response to calcium availability is rapid and leads to a reversible increase in raphide numbers (Franceschi, 1989). An increase in calcium availability also increases the number of raphides in callus tissue (Franceschi & Horner, 1979). The raphides are formed soon after cell division takes place and are diluted subsequently during expansion growth (Kausch & Horner, 1983; Tanaka et al., 2003a, 2003b). The raphides in all species are clumped together and surrounded by a mucilaginous sheath in the idioblast. The mucilage around the raphides stains positively for polysaccharides, is mainly extracted in the water and 2 M KOH fractions, and contains glucuronic acid, glucosyl, rhamnosyl, xylosyl, and mannosyl units and the N‐linked arabinogalactan and arabinans (Kausch & Horner, 1983; Paull et al., 1999; Webb et al., 1995).

The idea that raphides cause acridity in taro dates from at least the 1670s when needle‐shaped raphides were observed (Haberlandt, 1914; Leeuwenhoek, 1675; Pedler & Warden, 1888). Acridity in taro, other edible Aroids, and some other crops occurs in all parts of the plant with different intensities (Black, 1918; Bradbury & Nixon, 1998; Paull et al., 1999; Pedler & Warden, 1888; Sakai & Hanson, 1974). Acridity, besides being a quality problem in edible Aroids, is a problem for other crops such as kiwi fruit and some edible palm fruits (Bruynzeel, 1997; Julian & Bowers, 1997; Konno et al., 2014; Perera et al., 1990; Salinas et al., 2001; Walker & Prescott, 2003). Ornamental plants, such as Dieffenbachia, daffodils, and hyacinths, can irritate the skin of those who handle them and can pose health risks to children and pets because of acridity (Arditti & Rodriguez, 1982; Barnes & Fox, 1955; Coté, 2009; Gardner, 1994).

However, no direct evidence has shown that acridity is caused by mechanical puncturing of the skin and several studies suggest that the raphides do not cause acridity (Moy et al., 1979; Paull et al., 1999; Pohl, 1964). Evidence that acridity is due not to the raphides but to a factor on the raphides includes the following: (i) Acridity is lost with cooking and after extraction with methanol, ethanol, distilled water, and water/detergent mixtures with intact raphides remaining (Akpan & Umoh, 2004; Bradbury & Nixon, 1998; Chai & Liebman, 2005; Moy et al., 1979; Payne et al., 1941; Pedler & Warden, 1888; Saha & Hussain, 1983; Tang & Sakai, 1983; Tsai et al., 2006); (ii) there is no strong relationship between acridity and raphide number or amount of calcium oxalate extracted (Halloway et al., 1989; Moy et al., 1979; Payne et al., 1941); (iii) species that have pointed raphides can have very low or no acridity (Bradbury & Nixon, 1998; Ledbetter & Porter, 1970); (iv) the slowness of the acridity response at low concentrations does not suggest mechanical injury; (v) wide variation in sensitivity to taro acridity is not consistent with mechanical penetration; (vi) fewer raphide bundles are often found in the more acrid taro cultivars (Moy et al., 1979); and (vii) treatment of purified acrid raphides with protease leads to rapid loss of acridity with no change in raphide morphology (Paull et al., 1999). An alternate possibility is that chemical irritants or allergens occur on the surface of the raphides (Bradbury & Nixon, 1998; Konno et al., 2014; Nixon, 1987; Paull et al., 1999; Suzuki et al., 1975; Tang & Sakai, 1983), and the raphides play a synergist role as carriers (Konno et al., 2014).

Here we show that acridity is most likely due to an allergen protein associating with the raphides (Paull et al., 1999). The object of this research was to determine the cause of acridity and develop an assay for use by plant breeders and the food industry. To address this goal, we have attempted to determine whether peptides associated with purified raphides are potential allergens and whether the process of crystal formation is associated with this allergen.

## 2 MATERIAL AND METHODS

### 2.1 Plant material and raphide isolation

Acrid raphides were extracted from taro petioles sourced from the Poamoho Experiment Station on the Island of Oahu. Petiole bases were used to minimize contamination with starch. The few starch granules present in petiole extracts were also smaller than those found in the corm extract (Bradbury & Nixon, 1998; Paull et al., 1999). Fresh taro leaf petioles were chopped into small pieces and frozen at −20°C until needed. Frozen petiole pieces (150–160 g FM) were ground in petroleum ether (160 ml) for 1 min in a commercial blender at full speed; the top liquid was poured off quickly into a beaker and the solid squeezed with a spatula. An additional 140 ml petroleum ether was added to the solid and rehomogenized for 30 s, the liquid was combined with the previous collection, and the solid discarded. The liquid collected was covered and allowed to stand until the solids (crude raphides) settled, and the clear supernatant liquid was decanted. The solids were transferred with a minimum volume of petroleum ether to a Teflon centrifuge tube and allowed to settle again, with most of the clear petroleum ether being removed. An equal volume of chloroform was added, and the tube was vigorously shaken then centrifuged at 10,000*g* for 10 min, with the top layer discarded. The pellet in the bottom was washed three times with 2–5 ml of petroleum ether to produce an acrid raphide sample with very few starch granules. The purity of the raphides was checked by light microscope. The purified raphides were routinely stored at −20°C (Paull et al., 1999).

Purified raphides were washed with ether, dried, and suspended in aliquots of ReadyPrep Rehydration Buffer (8 M urea, 2% CHAPS, 50 mM dithiothreitol, .2% (w/v) Bio‐Lyte 3/10 ampholytes, and Bromophenol Blue). The suspension was boiled for 10 min, chilled in ice, and centrifuged at 10,000*g* for 1 min. The supernatant with dissolved protein was collected and raphides discarded. One hundred twenty‐five milliliters of sample buffer was applied to 7 cm of pH 4‐7 ReadyStrip IPG strip and isoelectric focusing performed with Bio‐Rad Protein IEF cell (Bio‐Rad). After focusing, the IEF strip was equilibrated and transferred onto a 15% (v/v) acrylamide gel, run at 150 V, and stained with Coomassie blue or silver to visualize peptides (Paull et al., 1999).

### 2.2 MALDI‐TOF‐MS of proteins from 2D gels

Protein spots were cut from the 2D gels and extracted with extraction buffer (100 mM NaHCO_3_, 8 M urea, 3% SDS, .5% Triton X‐100, 25 mM DTT) for 30 min at 65°C, crushed with a mortar and pestle, then allowed to stand overnight at 50–60°C. The slurry extract was transferred to a centrifugal separator tube, washed with 150 ml of 50% methanol, spun for 30 min at 13,000 g, washed with 400 ml wash buffer (2 M urea), recentrifuged, and vacuum dried. The 11 peptides in 2D gel spots of sufficient sample size that were considered to differ between highly acrid taro breeding variety 99‐6 and low‐acridity Chinese taro “Bin Liang” were forwarded to Harvard University Medical School Mass Spectrometry Facility for trypsin digestion, fractionation by high‐performance liquid chromatography (HPLC), and gas chromatography‐mass spectrometry (GC/MS) peptide sequencing. The peptide sequence data obtained from the 11 samples plus the corm sample were queried using BLASTp at NCBI (Altschul et al., 1997) and mapped to the taro genome (Yin et al., 2020).

### 2.3 Transcriptome sequencing

The upper corm and leaves of “Bin Liang” taro were harvested, the leaves and petioles were removed, and the apical meristems with the very young leaves and leaf primordia were excised. The tissue was frozen in liquid nitrogen and ground into powder. Total RNA was extracted with PureLink Plant RNA Reagent (Invitrogen, Carlsbad, California), treated with DNAse, and purified with the TruSeq stranded mRNA Sample Prep Kit, followed by standard Illumina single‐end sequencing. Illumina recommended protocols for sample purification; library preparation of cDNAs, fragmentation, adapter ligation, PCR amplification, and size selection were used. Sequencing was performed at the Roy J. Carver Biotechnology Center at the University of Illinois Urbana‐Champaign on a HiSeq2000 platform and using a TruSeq SBS sequencing kit (version 3).

After sequencing, a total of 236,711,776 (100 bp, single‐end) raw reads were obtained. FastQC (version v.0.11.9) was used to perform a quality control check on the sequencing output. Then low‐quality raw sequences were trimmed, and adapters were removed using Trimmomatic (version 3). Clean reads were aligned to the latest taro genome (“Taro_Lachesis_assembly_Chr.fa”; Yin et al., 2020) using Hisat2 (version 2.0.5). After alignment, sam files were sorted and converted to bam files using samtools (version 1.9). Reads that aligned to features were counted using featureCounts (Liao et al., 2014; version 2.0.0) and a gtf file obtained from the latest taro annotation file (“Taro_Chr_genome_all_transcripts_final_gene.gff3”; Yin et al., 2020). Finally, raw counts were manually converted to RPKM by calculating a “per million” scaling factor per library and then normalizing them by sequencing depth and gene length.

The assembled transcription sequences were mapped against the taro genome (Yin et al., 2021). Further annotation was performed with BLASTp at NCBI against Viridiplantae, SignalP‐5.0 (Armenteros, Salvatore, et al., 2019), TargetP‐2.0 (Armenteros, Tsirigos, et al., 2019), Probius (Käll et al., 2007), ApoplastP (Sperschneider et al., 2018), and DeepLoc‐1.0 (Armenteros et al., 2017).

### 2.4 Network analysis

The gene co‐expression network was inferred using GWENA (Lemoine et al., 2021). This software is a modified version of WGCNA (Langfelder & Horvath, 2008) that includes methods for visualizing co‐expression networks, network modules, hub gene detection, and differential co‐expression. The network was visualized within R (R Core Team, 2021), and the weight cutoff was set at *p* < .01. The resulting network is a scale‐free weighted gene network with multiple nodes representing genes and connected by edges.

### 2.5 cDNA library construction and cDNA cloning

Apical meristem tissues from “Bin Liang” was excised as described above, frozen in liquid nitrogen, and ground into powder, and total RNA was isolated with the PureLink Plant RNA Reagent (1 g in 10 ml) (Invitrogen Life Technologies, Carlsbad, California). Poly‐A RNA was isolated from total RNA using NucleoTrap mRNA MiniKit (Clontech Laboratories Inc., Mountain View, California). cDNA library was constructed with SMART cDNA Library Construction Kit (Clontech Laboratories Inc., Mountain View California). The library was synthesized by Long‐Distance PCR method due to the low amount of mRNA isolated.

The GeneRacer Kit (L150201, Invitrogen Life Technologies, Carlsbad, California) was used to obtain full‐length cDNA clones of profilin, actin, and ATPase that were sequenced with 5′‐and 3′‐end, gene‐specific primers designed for most common sequences based upon transcriptional analysis. None of the primers in the 5′ region yielded a profilin PCR product; only primers located on the 3′ end of the sequence yielded positive profilin clones. Nested PCR was performed to eliminate nonspecific amplification and increase sensitivity and specificity, and the resulting single bands at 700 and 500 bp were isolated and cloned. A ligated PCR product synthesized with new primers was identified as a profilin clone.

### 2.6 Allergenicity queries

Allergenicity database at the University of Nebraska, Allergen Protein Database (http://www.allergenonline.org/) (Goodman et al., 2016) was used to query our assembled peptides and transcriptome sequences against known allergens. B‐cell epitope predictions were made with BepiPred‐2.0 (Jespersen et al., 2017) that identifies epitopes annotated from antibody–antigen protein structures. T‐cell epitopes were evaluated with the TepiTool (Paul et al., 2016) at IEDB (Fleri et al., 2017) that predicts peptide binding to major histocompatibility complex (MHC) receptors (i.e., antigenicity but not immunogenicity).

### 2.7 Profilin antibodies to raphide 2D western blot and rapid allergen testing

Polyclonal antibodies from goat to *Arabidopsis thaliana* profilin (Santa Cruz Biotechnology, Dallas, Texas, SC‐15949) were used to detect profilin in 2D gels of taro raphides by western blotting following the manufacturers protocol. Neogen's Reveal 3‐D for Hazelnut test kit (Neogen, Lexington KY, USA) is a rapid ELISA assay for hazelnut allergens on environmental swabs, rinses and foods. Taro leaves were pressed and liquid collected. An equal volume of extraction buffer was added to the taro leaf liquid and mixed for 1 min. The Reveal 3‐D device was dipped in the liquid for 1 min, and banding was allowed to develop and checked after 5 min.

## 3 RESULTS

### 3.1 Raphide‐associated proteins

Ten of the 11 peptides from the acrid taro petiole and corm 2D gel separation (supporting information Figure S1) gave multiple peptide sequences (Table 1). One sample gave only one useable peptide sequence (GILYLGMGVSGGEEGAR) that mapped to EVM0003651 and was 96% homologous to 6‐phosphogluconate dehydrogenase, decarboxylating 1 isoform X1. A number of the MS peptide sequences mapped to different gene alleles in the taro genome (supporting information Table S1). Raphide sample spot #5 gave two peptide sequences that mapped to four predicted genes that had high homology to profilin at a molecular weight of about 30 kDa, while sample #23 had homology to profilin with an expected profilin molecular weight of 15–16 kDa. All four predicted profilin genes were expressed in the taro apex (Table 2). Raphide‐associated peptide sequences gave high sequence identities in BLASTp searches with greater than 83% similarity (Table 1). The peptides predicted from the corm 2D gel were more diverse and showed homology with five different proteins with varying molecular weights (Tables 1 and 2). The pattern of spots on 2D gels for a taro cultivar that is regarded as having less acridity showed fewer peptides (supporting information Figure S1) in the profilin‐associated areas.

**TABLE 1:** Eleven of the 28 proteins separated from the purified raphides from petioles and corms by 2D gels electrophoresis (supporting information Figure S1) then subjected to peptide GC/MS peptide sequencing and mapped to the taro genome (Yin et al., 2020)

2D‐gel spot GC/MS sequences	Potential gene	Description	Length	e‐value	Similarity
Raphide 1	EVM0027136	ATP synthase subunit beta, mitochondrial	329	0	99.97
Raphide 1	EVM0027796	ATP synthase subunit beta, mitochondrial	286	0	89.58
Raphide 2	EVM0016966	ATP synthase subunit beta, mitochondrial	555	0	93.29
Raphide 2	EVM0027136	ATP synthase subunit beta, mitochondrial	329	0	99.97
Raphide 2	EVM0027796	ATP synthase subunit beta, mitochondrial	286	0	89.58
Raphide 3	EVM0006413	V‐type proton ATPase subunit B 2 isoform X2	485	0	99.64
Raphide 3	EVM0017279	V‐type proton ATPase subunit B 2 isoform X2	480	0	99.81
Raphide 4	EVM0010466	Hypothetical protein	378	0	99.55
Raphide 4	EVM0009495	Actin	378	0	99.95
Raphide 4	EVM0021925	Actin‐101‐like protein	378	0	99.58
Raphide 4	EVM0022292	Actin	378	0	99.81
Raphide 4	EVM0023828	Actin	378	0	99.87
Raphide 5	EVM0003866	Profilin, putative	150	1.42E‐88	82.89
Raphide 5	EVM0004262	Profilin, putative	133	3.46E‐85	91.87
Raphide 5	EVM0017269	Profilin, putative	133	5.15E‐89	92.75
Raphide 5	EVM0021119	Profilin, putative	135	1.33E‐83	88.46
Paphide 11	EVM0000630	Chaperonin subunit beta 60 kD	606	0	94.96
Raphide 11	EVM0016966	ATP synthase subunit beta, mitochondrial	555	0	93.29
Raphide 11	EVM0027136	ATP synthase subunit beta, mitochondrial	329	0	99.97
Raphide 11	EVM0027796	ATP synthase subunit beta, mitochondrial	286	0	89.58
Raphide 12	EVM0016966	ATP synthase subunit beta, mitochondrial	555	0	93.29
Raphide 12	EVM0027136	ATP synthase subunit beta, mitochondrial	329	0	99.97
Raphide 12	EVM0027796	ATP synthase subunit beta, mitochondrial	286	0	89.58
Raphide 21	EVM0009495	Actin	378	0	99.95
Raphide 21	EVM0010466	Actin	378	0	99.55
Raphide 21	EVM0019011	Actin	378	0	99.92
Raphide 21	EVM0021925	Actin‐101‐like protein	378	0	99.58
Raphide 21	EVM0022292	Actin	378	0	99.81
Raphide 21	EVM0023828	Actin	378	0	99.87
Raphide 21	EVM0022724	ATP synthase subunit alpha, mitochondrial	493	0	94.44
Raphide 22	EVM0003651	Phosphogluconate dehydrogenase decarboxylating	496	0	91.34
Raphide 23	EVM0003866	Profilin, putative	150	1.42E‐88	82.89
Raphide 23	EVM0004262	Profilin, putative	133	3.46E‐85	91.87
Raphide 23	EVM0017269	Profilin, putative	133	5.15E‐89	92.75
Raphide 23	EVM0021119	Profilin, putative	135	1.33E‐83	88.46
Corm Raphide 1	EVM0000630	Chaperonin subunit beta 60 kD	606	0	94.96
Corm Raphide 2	EVM0004708	MENTAL domain‐containing protein	270	7E‐122	71.15
Corm Raphide 5	EVM0009495	Actin	378	0	99.95
Corm Raphide 6	EVM0010466	Actin	378	0	99.55
Corm Raphide 7	EVM0018066	RNA polymerase II transcription subunit 37c	649	0	97.07
Corm Raphide 8	EVM0019011	Actin	378	0	99.92
Corm Raphide 9	EVM0021925	Actin‐101‐like protein	378	0	99.58
Corm Raphide 10	EVM0022292	Actin	378	0	99.81
Corm Raphide 12	EVM0022414	Heat shock cognate 70 kDa protein 2‐like	462	0	99.14
Corm Raphide 13	EVM0022454	Heat shock cognate 70 kDa protein	645	0	97.84
Corm Raphide 14	EVM0027226	RNA polymerase II transcription subunit 37c	649	0	98.12
Corm Raphide 15	EVM0022292	Actin	378	0	99.81
Corm Raphide 16	EVM0023828	Actin	378	0	99.87

*Note*: The predicted gene sequences were subjected to BLASTp, and their description and homology were determined. Each peptide spot on the 2D gel represented comigration of the product from more than one gene. Location of peptide spots are shown in the supporting information Figure S1.

Abbreviation: GC/MS, gas chromatography‐mass spectrometry.

**TABLE 2:** The characteristics of the gene products predicted from the mass spectrometry (MS) peptide sequences and mapped to the taro genome, their molecular weight, the number of residues, their charge, expression as RPKM in taro apex and standard error (SE) of mean, result of allergen prediction (SDAP), and potential cellular targeting (DeepLoc)

Gene	Description	Molecular weight	Number amino acids	Predicted pI	Total number negatively charged (Asp + Glu)	Total number positively charged (Arg + Lys)	Apex RPKM expression mean	Apex expression SE *n* = 3	SDAP allergen	DeepLoc location
EVM0010466	Actin	41,637	377	5.3	50	38	1280.05	124.57	No	Cytoplasm
EVM0022292	Actin	41,707	377	5.3	50	38	232.27	27.12	No	Cytoplasm
EVM0009495	Actin	41,669	377	5.3	50	38	379.03	32.33	No	Cytoplasm
EVM0023828	Actin	41,687	377	5.31	50	38	0.01	0.01	No	Cytoplasm
EVM0019011	Actin	41,739	377	5.3	50	38	65.28	4.35	No	Cytoplasm
EVM0023003	Actin	41,697	377	5.31	50	38	0.00	0.00	No	Cytoplasm
EVM0023828	Actin	41,687	377	5.31	50	38	0.01	.01	No	Cytoplasm
EVM0021925	Actin‐101‐like protein	41,693	377	5.32	50	38	14.98	2.14	No	Cytoplasm
EVM0008510	Actin‐like protein	41,661	377	5.3	50	38	0.00	.00	No	Cytoplasm
EVM0022724	ATP synthase subunit alpha, mitochondrial	53,531	492	5.86	57	52	132.80	38.49	No	Cytoplasm
EVM0027796	ATP synthase subunit beta, mitochondrial	30,682	286	5.73	32	27	101.19	7.94	No	Cytoplasm
EVM0027136	ATP synthase subunit beta, mitochondrial	35,245	328	5.84	36	31	30.16	1.62	No	Cytoplasm
EVM0016966	ATP synthase subunit beta, mitochondrial	59,016	554	6.08	62	58	248.82	23.56	No	Mitochondrion
EVM0009509	ATP synthase subunit beta, mitochondrial	45,017	395	6.57	41	37	0.00	0.00	No	Cytoplasm
EVM0014402	ATP synthase subunit beta, mitochondrial	51,178	479	8.41	46	48	0.00	0.00	No	Mitochondrion
EVM0000630	Chaperonin 60 kDa subunit beta	64,438	605	5.66	83	78	137.88	19.43	No	Plastid
EVM0005930	Chloroplastic Mn‐stabilizing protein 1	34,628	330	5.87	38	37	0.00	0.00	No	Plastid
EVM0028022	Heat shock cognate 70 kDa 2‐like	71,092	648	5.2	99	82	0.00	0.00	No	Cytoplasm
EVM0003651	Phosphogluconate dehydrogenase, decarboxylating 1	59,588	540	6.19	68	65	0.00	0.00	No	Plastid
EVM0021119	Profilin, putative	14,386	134	6.05	15	13	.35	0.18	Yes	Cytoplasm
EVM0004262	Profilin, putative	13,908	132	4.51	17	8	52.51	3.83	Yes	Cytoplasm
EVM0003866	Profilin, putative	15,916	149	6.4	15	14	375.18	17.74	Yes	Cytoplasm
EVM0017269	Profilin, putative	13,990	132	4.73	16	8	299.90	14.98	Yes	Cytoplasm
EVM0015886	Profilin, putative	14,062	132	5	14	9	0.00	0.00	Yes	Cytoplasm
EVM0022454	RNA polymerase II transcription mediator subunit 37c	70,944	645	5.17	101	82	0.02	0.01	No	Cytoplasm
EVM0006413	V‐type proton ATPase subunit B 2 X2	54,077	484	5.17	67	52	53.01	4.04	No	Cytoplasm
EVM0017279	V‐type proton ATPase subunit B 2 X2	53,519	479	5.12	66	51	237.50	3.69	No	Lysosome/vacuole

### 3.2 Gene characterization

The 27 genes predicted from the MS peptide sequences were 11 actins, one ATP synthase alpha subunit and five ATP synthase beta subunits, one chloroplastic 60 ‐kDa chaperonin subunit beta, one chloroplastic manganese‐stabilizing protein 1, one heat shock cognate 70‐kDa protein 2‐like, one 6‐phosphogluconate dehydrogenase, decarboxylating 1 isoform X1, five profilins, one RNA polymerase II subunit, and two V‐type proton ATPase B2 subunits (Table 2). The sequences varied in pI from 4.5 for one of the profilins to 8.4 for a beta subunit of ATPase synthase, with most between 5 and 6.5. The greatest variations in the profilin amino acid complement were in arginine, isoleucine, proline, and threonine (supporting information Table S2). All the profilins had two cysteine residues (supporting information Table S6) at C^13^ and C^115^ (or C^132^ for EVM0003866 with 17‐amino acid insert). Only profilin was flagged as a potential allergen (Table 2). TargetP‐2.0 and DeepLoc‐1.0 predicted mitochrondrial and chloroplast targeting sequences on two of the three beta ATPase subunits and the two chloroplastic products (EVM0000630, EVM0005930) (Table 2). SignalP, Phobius, and ApoplastP did not show any targeting peptides to any of these gene products.

### 3.3 Apex transcription expression

**FIGURE 1:**
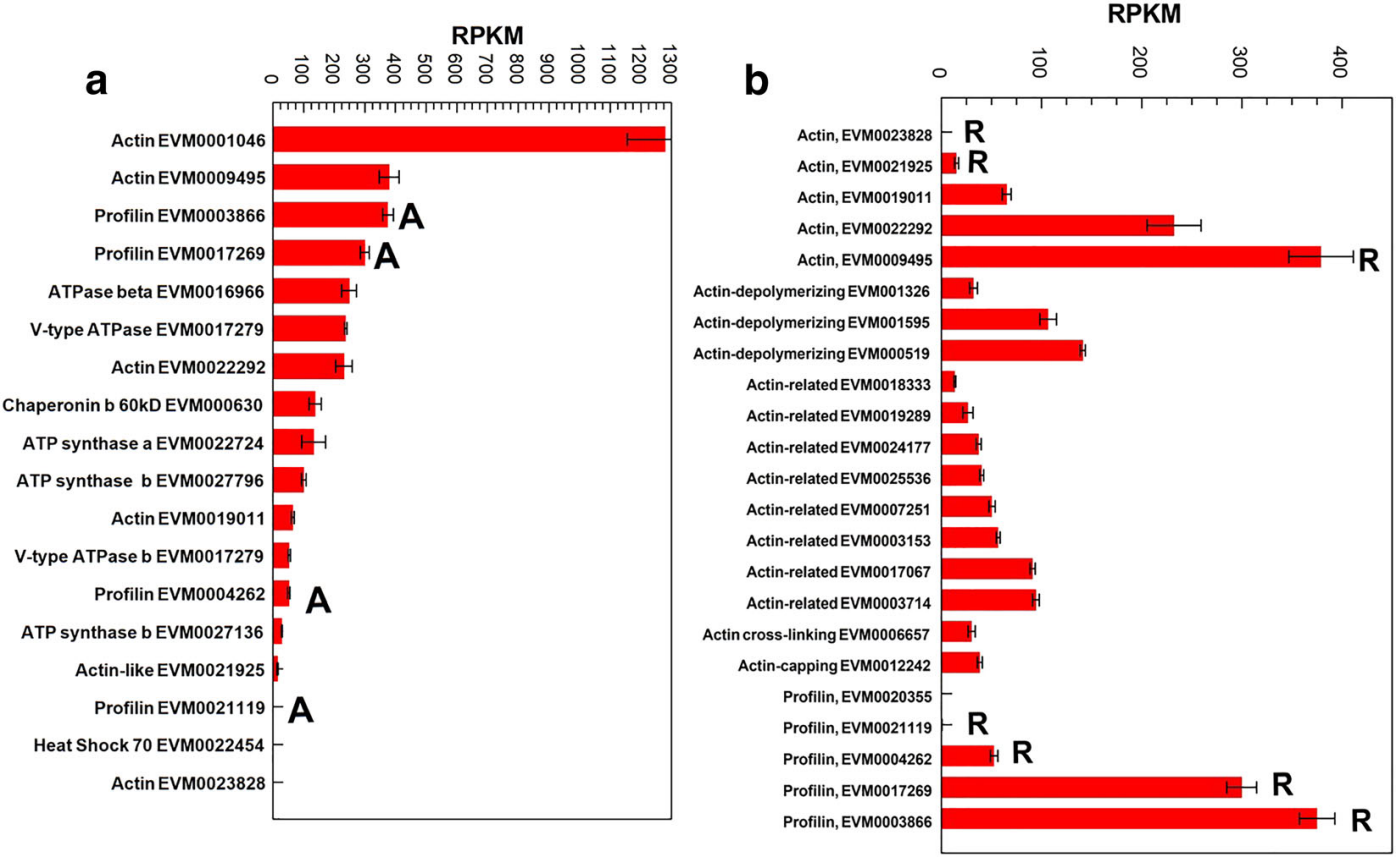
Gene expressed in the taro apex associated with the raphides predicted peptides from mass spectrometry (MS) sequencing and apex transcription analysis. (a) Raphide genes, bars topped with “A” predicted to allergens and (b) actin and actin‐related peptides in taro apex, bars marked with “R” were found in the raphide MS analysis. Other expressed cytoskeletal related genes are given in the supporting information Table S7. Mean + SE, *n* = 3

**FIGURE 2:**
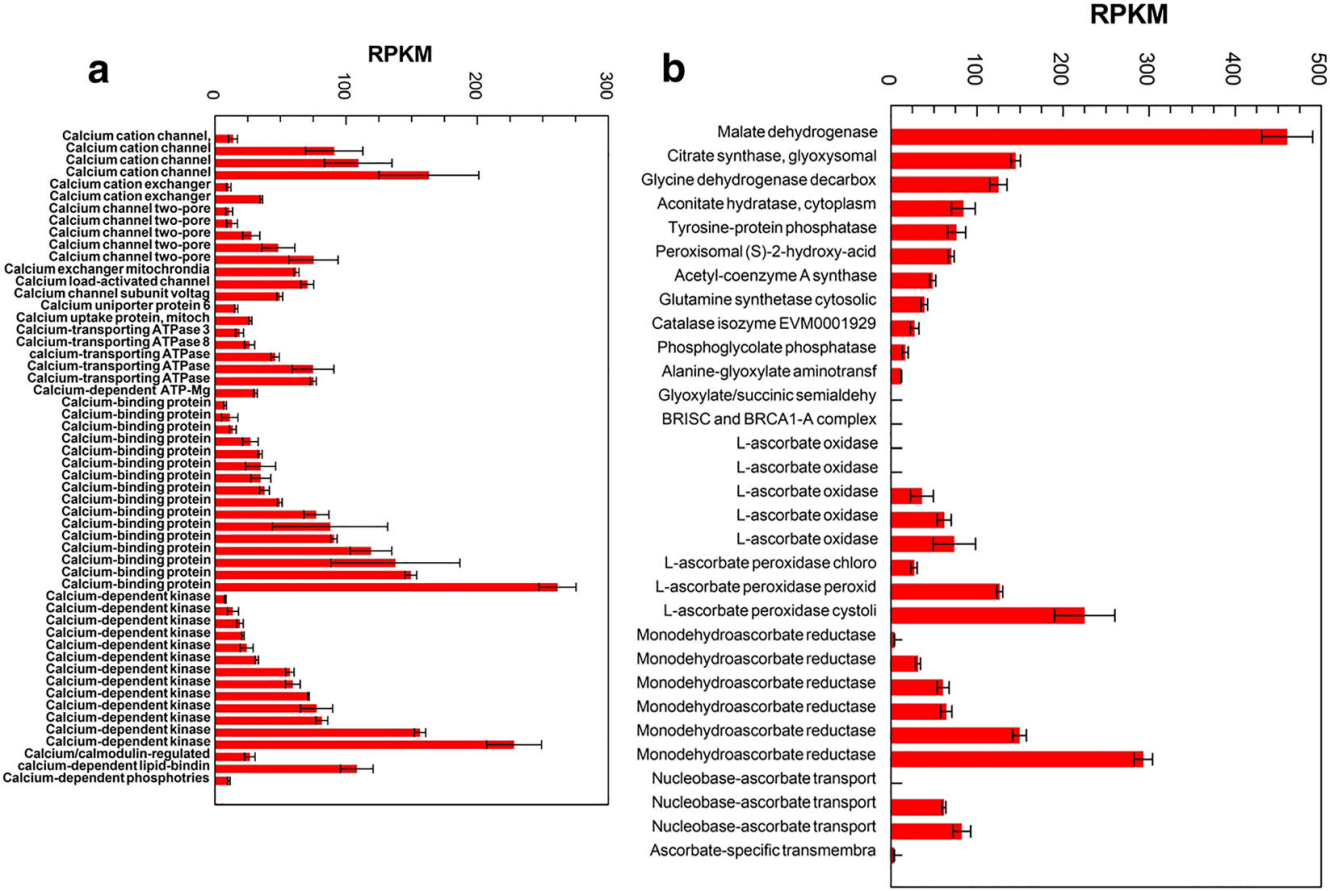
Taro apex genes expressed and potentially involved in (a) calcium metabolism, transport, Ca‐dependent kinases and Ca‐binding and (b) genes expressed in the apex with potential roles in oxalate metabolism. Mean + SE, *n* = 3

The 27 genes predicted from the MS peptide sequences analysis showed variable expression in the taro apex (Table 2). The 18 genes expressed are dominated by actin and profilin (Figure 1a). Four out of the five actin and actin‐related genes were raphide‐associated (Figure 1b). Calcium cation channels and uniporters, Ca‐transporting ATPase with calcium‐binding proteins and calcium‐dependent protein kinases, and calmodulin were expressed (Figure 2a). None were found associated with or bound to the raphides. Numerous genes potentially involved in oxalate synthesis were found expressed (Figure 2b). These oxalate‐related genes were associated with ascorbic acid and glycolate metabolism and with the Krebs cycle.

### 3.4 Apex transcriptome expression network analysis

**FIGURE 3:**
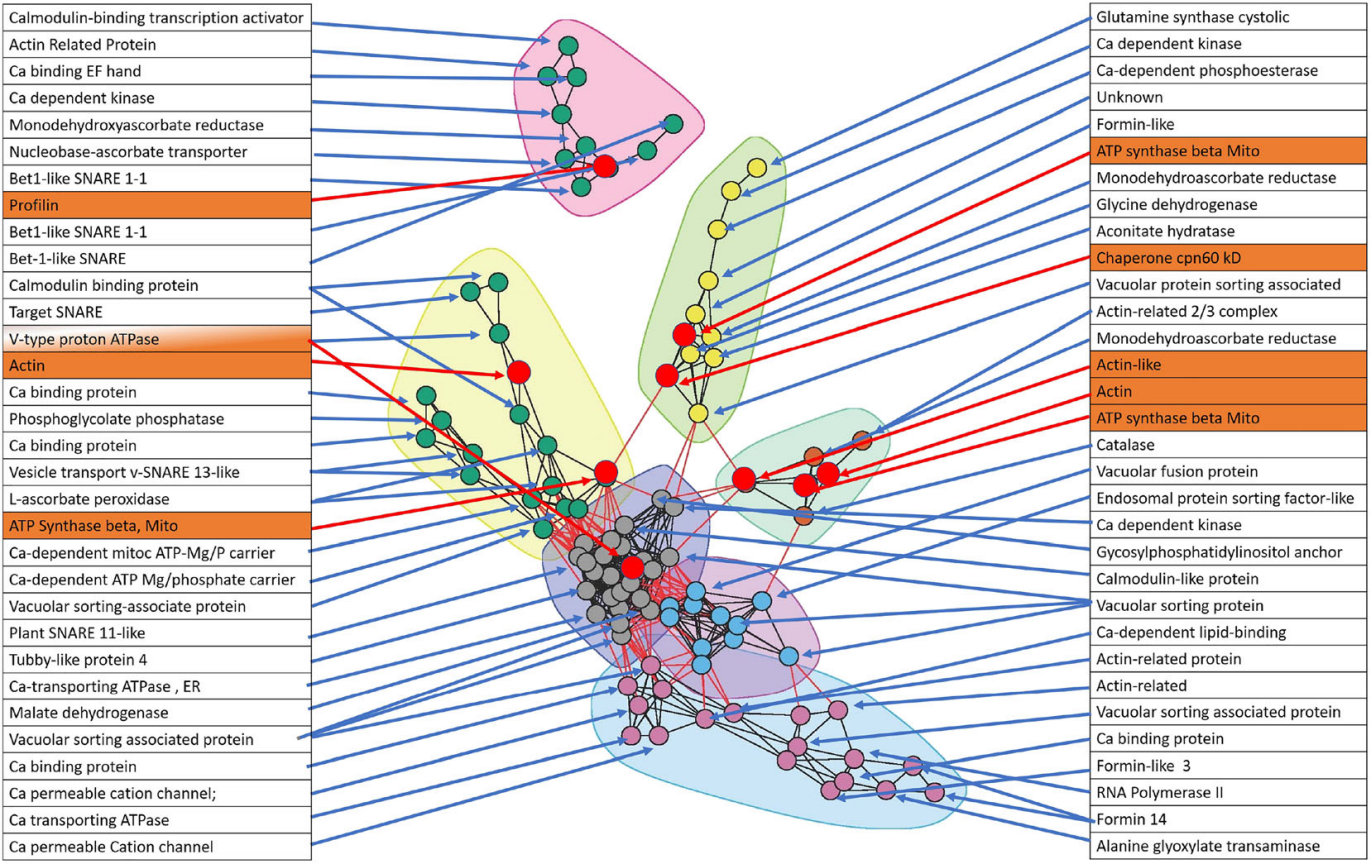
Gene network showing eight interconnected clusters of genes. Genes highlighted in red were those genes predicted to be on the mature raphides. Network genes included in the analysis were those associated with the raphides, cytoskeleton, oxalate metabolism, endosomal, vesicle, and membrane fusion and transport. The partial correlation coefficients were all greater than +0.99. The genes found in the dense gray central cluster are given in the supporting information Table S4

Since the raphide‐associated peptides were predicted to be from different organelle sources, network analysis was performed on expressed genes found on the raphides, associated with cytoskeletal elements, calcium‐related activities, oxalate metabolism and predicted involvement in vesicular transport, and fusion. Network analysis gave six interconnected clusters (Figure 3) connected to raphide‐associated genes with partial correlation coefficients greater than .990 (supporting information Table S4). Three of the clusters had edge connection to other clusters of genes predicted to be on the taro raphides. One cluster was dominated by actin or actin‐related genes, ATPase beta subunit, catalase, and monodehydroascorbate reductase. Monodehydroascorbate reductase is involved in ascorbate biosynthesis and oxalate metabolism. Three other clusters were connected via genes whose products were found on mature taro raphides: ATPase B‐subunit (EVM0016966), V‐type proton ATPase Beta subunit (EVM0006413), and a chaperone cpn 60 kDa (EVM0000630). Many of the other genes in the clusters were related to calcium, ion channels, and protein kinases. These genes were networked to ascorbate, citrate, and malate metabolism and linked to actin (formin, actin depolymerization) genes. The dense central cluster of 25 genes (supporting information Table S4, Cluster #2) was dominated by genes involved in the Golgi network and vesicle transport, protein targeting, anchoring, calcium‐binding, and a V‐Type proton ATPase (EVM0006413) found on the raphides. The adjoining dense cluster (#16) was dominated by genes with homology to protein targeting to the vacuole, vacuolar transport and fusion, endosomal sorting, actin depolymerization, and a phosphatidylinositol kinase. A separate unconnected cluster (Figure 3) included one of the profilin genes (EVM0004262), while other profilin raphide‐associated genes were found in single gene clusters.

### 3.5 Profilin characterization

**FIGURE 4:**
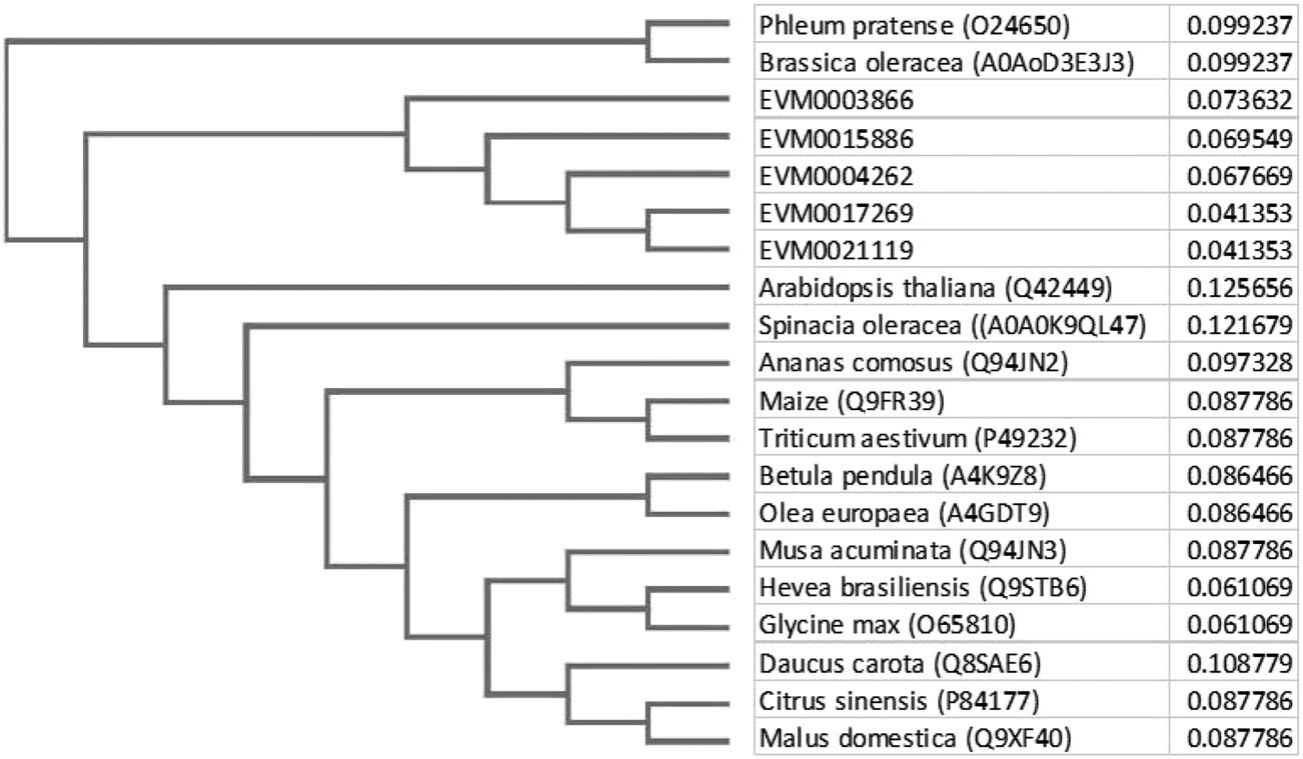
Clustal W Phylogram of the five predicted profilin genes from taro with selected profilins from other species some of which have not been reported to cause an allergenic reaction. Full gene alignment is given in the supporting information Table S5

Since only the profilins were predicted to be allergens, we focused on their structural characterization. Five predicted taro profilin genes cluster together in Clustral‐W Phylogram, with EVM0003866 being more distantly related, though all taro profilins cluster separately from other species (Figure 4). The 17‐amino acid insert sequence in EVM0003866 had little homology (BLASTp) with other higher plant sequences with e‐values being .034, 68% identity. In addition to seven β‐sheets between the N‐ and C‐terminals, the profilins had three α‐helixes, including one at either end, with the longest α‐helix at the C‐terminal end and the α‐helix bracketed by two β‐sheets on one side and five on the other (supporting information Table S6).

All five taro profilins had motifs that would impart interactions with monomeric actin, proline‐rich proteins, and phosphatidylinositol bisphosphate lipids. Motifs for actin A^64^, P^65^, Q^79^, V^85^, R^87^, K^89^, K^90^, T^114^, P^115^, G^116^, N^119^, M^120^, R^124^ (Jimenez‐Lopez et al., 2013; Schluter et al., 1998; Thorn et al., 1997); proline binding W^3^, Y^6^, I^28^, G^30^, W^36^, A^37^, Y^128^, L^129^ (Lambrechts et al., 2002; Thorn et al., 1997); and phosphatidylinositol bisphosphate W^3^, D^8^, K^74^, K^89^, G^91^, M^120^, L^130^, E^131^ (Fedorov et al., 1994; Skare & Karlsson, 2002) were in agreement allowing for the taro's amino acid insertions and some substitutions. The amino acid sequence, electrostatic potential (hydrophobicity), binding motifs, and predicted taro profilin structure suggest strongly conserved and generally similar 3D folding in common with other plant profilins.

**FIGURE 5:**
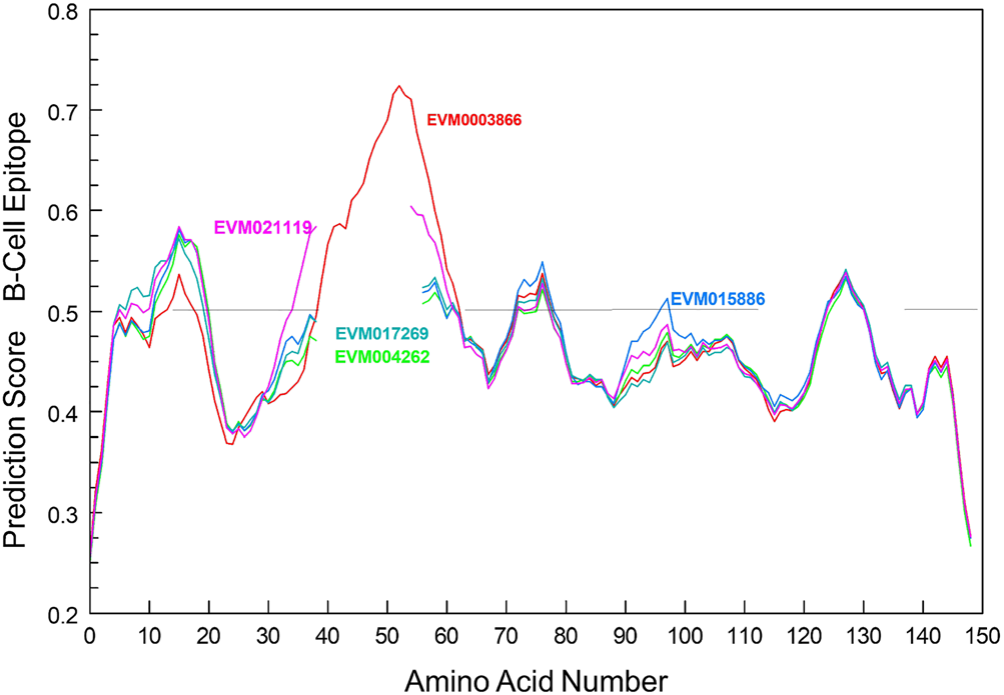
Alignment of taro's five profilins showing the high antigenicity areas (Jespersen et al., 2017) using BepiPred‐2.0 trained on epitopes and nonepitope amino acids determined from crystal structures, with predicted B‐cell epitopes greater than a threshold of 0.5. B‐cell epitope showing the five sections of the profilin sequence with greater than the default threshold of 0.5 and increase in the epitope score of the second epitope when the 17 amino acids insert that occurred in EVM0003866

Taro profilin gene sequences did retain high homology to *Betula pendula*, or hazelnut, in the areas of the peptide with high antigenicity (supporting information Table S5). Of the seven areas in hazelnut with high antigenicity, two areas in taro are 100% identical, with one or more areas of the peptide having high similarity. The second antigenic epitope in hazelnut (DGQGQQLA) and other plant profilins have little similarity to EVM0003866 that had the significant insert of 17 amino acids, and EVM0021119, with two amino acids that both enhanced the second profilin B‐cell epitope (Figure 5). The EVM0003866 increased the B‐cell epitope score from .534 to .724, a 35% increase and specificity of .99954 (Jespersen et al., 2017), while EVM0021119 went to .604. Structural analysis predicted that these were coiled sections that were exposed on the folded peptide (supporting information Table S6). All five taro profilins had peptide sequences that could also bind to human MHC class I and class II with overlap with B‐cell epitopes, implying T‐cell antigenicity but not necessarily immunogenicity, that is, ability to induce humoral and/or cell‐mediated immune response.

### 3.6 Isolation of clones from cDNA library

Twenty‐two profilin, one actin, and one ATPase cDNA were isolated and sequenced from our taro cDNA library (supporting information Table S3). It was our intent to use these clones to support the transcription sequencing results generated from Illumina short read sequencing and potentially express the genes in a heterologous expression system. The profilin clones isolated from cDNA library ranged from 780 to 850 nucleotides in length (132 amino acids) and were shorter than the profilin de novo assembled from the transcriptome, which had 1365 nucleotides. None of the profilin cDNA clones had the 17‐amino acid insert (EVM 0003866) or the 2‐amino acid insert (EVM0021119).

### 3.7 Cytoskeleton, endosome, and autophagy‐related gene expression

Cytoskeleton‐related genes in addition to actin, including formin, myosin, NET genes, nexin, SCAR (SCARCROW), VACUOLELESS, and villin were expressed in the taro apex (supporting information Table S7). These were not found in the sequenced raphide peptides but could have been present in faint 2D spots that were not sequenced. Genes associated with vacuolar transport and sorting, endosome membranes, and phosphatidylinositol kinase were expressed in the taro apex and could be involved in autophagy as inferred in the network analysis (supporting information Table S8).

### 3.8 Cross‐reactivity with commercial antibodies

The western blots were conducted with protein extracted from raphides of “Bin Liang,” a commercial cultivar with low acridity. Taro proteins were separated on 2D gel blot and probed with profilin polyclonal antibodies for *Arabidopsis thaliana* profilin. No profilin was detected. Taro profilins have only 58.5% to 75.8% identity to Arabidopsis.

Liquid from taro leaves was diluted with an extraction buffer, and the Neogen ELISA hazelnut device was dipped into the diluted sample. A faint positive band was observed after 5 min (Figure 5), suggested cross‐reaction with a protein epitope on a taro leaf component, as the test was made with polyclonal antibodies to hazelnut, for which profilin is a secondary allergen.

## 4 DISCUSSION

### 4.1 Identifying raphides as a putative allergen

Inorganic calcium oxalate crystals were among the earliest structures observed in plants (Leeuwenhoek, 1675), are widely observed in all major plant taxonomic groups (Franceschi & Nakata, 2005; McNair, 1932; Zindler‐Frank et al., 2001), and as with biominerals in other phyla have evolved independently and convergently (Gilbert et al., 2022). The crystals are found in the vacuoles of modified parenchyma cells called idioblasts in all plant organs (Arnott & Pautard, 1970; Foster, 1956; Franceschi & Nakata, 2005; Gallaher, 1975; Horner et al., 2012; Horner & Wagner, 1995; Nakata, 2003; Tang & Sakai, 1983). The proposed roles of these crystals include cellular calcium regulation, plant defense against herbivores, detoxification of aluminum, or a combination of these functions (Franceschi & Nakata, 2005; Karabourniotis et al., 2020; Nakata, 2012; Nakata, 2015). These crystals (druse, raphides, and sand) (Haberlandt, 1914; Sakai et al., Franceschi & Horner, 1980) have intrigued plant biologists leading to some limited use in taxonomy and systematics (Horner et al., 2012; Prychid & Rudall, 1999), as examples of biomineralization, and as objects of interest, especially the uniquely shaped raphides (Horner & Whitmoyer, 1972; Kausch & Horner, 1983, 1984; Mollenhauer & Larson, 1966; Parameswaran & Schultze, 1974; Sakai & Hanson, 1974; Tilton & Horner, 1980; Horner et al., 2000; Nakata, 2003, 2012). The Leeuwenhoek (1675) observations were of the leaf and sap of Arum plant in the family Araceae (Aroids) of which all genera are now regarded as toxic due to raphides (Burrows & Tyrl, 2001; Mrvos et al., 1991; Watson et al., 2005). However, the evidence for raphides causing acridity is circumstantial and based on their barbed needle appearance. Raphides might play an unwitting role as carriers and have a synergistic interaction with the acridity factor (Haberlandt, 1914; Konno et al., 2014)

Leeuwenhoek (1675) chewed the Arum leaves and concluded that what he called “pipes” from his microscopy observations were the cause of the “smart” taste (Late Old English ‐ “cause sharp pain”) and that the “pipes” were of firmer matter after exposure to fire. He also found these “pipes” in grapes, asparagus, spurge, and white hellebore. The description matches the bundles of raphides found in many plants and that these in some cases can cause the “smart” taste (acridity). The acceptance of raphides as responsible for acridity has focused research on biomineralization aspects including the synthesis, localization, and formation of the raphides. Efforts to show a relationship between the amount of calcium and oxalate extracted or raphide number and acridity have been unsuccessful (Halloway et al., 1989; Moy et al., 1979; Payne et al., 1941; Tang & Sakai, 1983). In addition, species that have raphides can have low or no acridity, including some varieties of taro (Paull et al., 2000), giant swamp taro (*Cyrtosperma merkusii*), Lemna spp., and Spirodela spp. (Bradbury & Nixon, 1998; Konno et al., 2014; Ledbetter & Porter, 1970; Tang & Sakai, 1983). Acridity varies widely across taro cultivars (Lebot et al., 2004; Paull et al., 2000), and people vary in their sensitivity (Watson et al., 2005). The focus on crystal formation has not led to an assay for this antinutrient component, and the absence of an assay has been a major limitation in breeding for reduced acridity (Lebot et al., 2004) and for processing of harvested material (Moy et al., 1979). In our earlier work (Paull et al., 1999), we provided evidence that the acridity was due to something on the raphides that were unstable and rapidly lost upon protease treatment. This followed from earlier research that questioned the raphides themselves being the cause of acridity (Halloway et al., 1989). Our early raphide‐associated peptide sequencing results suggested that the allergen‐like compound was a protease, but we did not see the same peptide sequence in this study (Table 1). Failure to detect the possible cysteine protease could be due to differences in the species being studied (Dieffenbachia vs. Colocasia), the 2D gel spots being sequenced, or could reflect contamination of the raphides with a vacuolar cysteine protease during extraction (Müntz, 2007). Konno et al. (2014) also suggested a possible cysteine protease involved with the raphides from kiwi fruit might cause mechanical holes in cell barriers. The difference in acridity between taro types may reflect differences in expression of l‐ascorbate oxidase and mitochondrial‐like calcium uniporter (Yin et al., 2020), with cytoskeleton genes not being differentially expressed.

### 4.2 Raphide‐associated peptides: A new potential allergen

Our raphide‐associated peptide sequencing detected two different areas on our 2D gels (Table 1) that had homology to profilin, with others spots matching ATPase (mitochondrial and vacuolar), ATP synthase, actin and a chaperonin (Figure 1a). The two spots (#5 and #23) that had homology to profilin had molecular weights of 15 and 30 kDa, respectively, while most profilins have molecular weights of 12 to 15 kDa (Davey & Moens, 2020; Santos & Van Ree, 2011). The spot at 30 kDa was potentially a dimer as has been reported for human (Babich et al., 1996) and maize profilins (Davey & Moens, 2020; O'Malley et al., 2021; Psaradellis et al., 2000). All the gene sequences for the predicted raphide‐associated peptides were submitted to Structural Database of Allergenic Proteins (SDAP). Only profilins were returned as being allergenic, matching the secondary allergens in hazelnuts and other nuts, birch pollen and latex, and being regarded as a pan‐allergen, with 20% of all pollen‐allergic patients displaying IgE reactivity to various plant profilins (Davey & Moens, 2020; Psaradellis et al., 2000; Santos & Van Ree, 2011; Valenta et al., 1992). Profilins are seen as minor allergens when compared with prolamins (seed storage proteins) and Bet v 1 (a pathogenesis‐related protein) (Jenkins et al., 2005). The three‐dimensional conserved structure despite low sequence similarity (Jimenez‐Lopez et al., 2013) has been determined for a number of profilins from plants (Fedorov et al., 1997; Thorn et al., 1997), with birch pollen profilin having a central six‐stranded antiparallel β‐sheet and two α‐helices that fold into a compact structure. This structure is similar to that predicted for the taro profilins with three α‐helixes (supporting information Table S6). The amino acids that contribute to linear and conformational epitopes in profilin and that take part in T‐ and B‐cell epitope binding can vary, as detected in the same and different olive cultivars (Jimenez‐Lopez et al., 2013). Linear B‐cell epitopes (Figure 5; supporting information Table S6) were predicted using BepiPred‐2.0 (Jespersen et al., 2017) based upon an algorithm trained on epitopes annotated from antibody–antigen protein structures. In 2D gels, taro profilins showed two molecular sizes suggesting dimers that have been shown to have greater immunogenicity than monomers (Psaradellis et al., 2000). Four profilin genes were identified and expressed in our taro apex transcriptome analysis, while the remaining taro profilin (EVM15886) was not expressed. All taro profilins are predicted to contain conserved actin, proline, and phosphatidylinositol 4, 5‐bisphosphate‐binding motifs (Jimenez‐Lopez et al., 2013; Krishnan & Moens, 2009).

**FIGURE 6:**
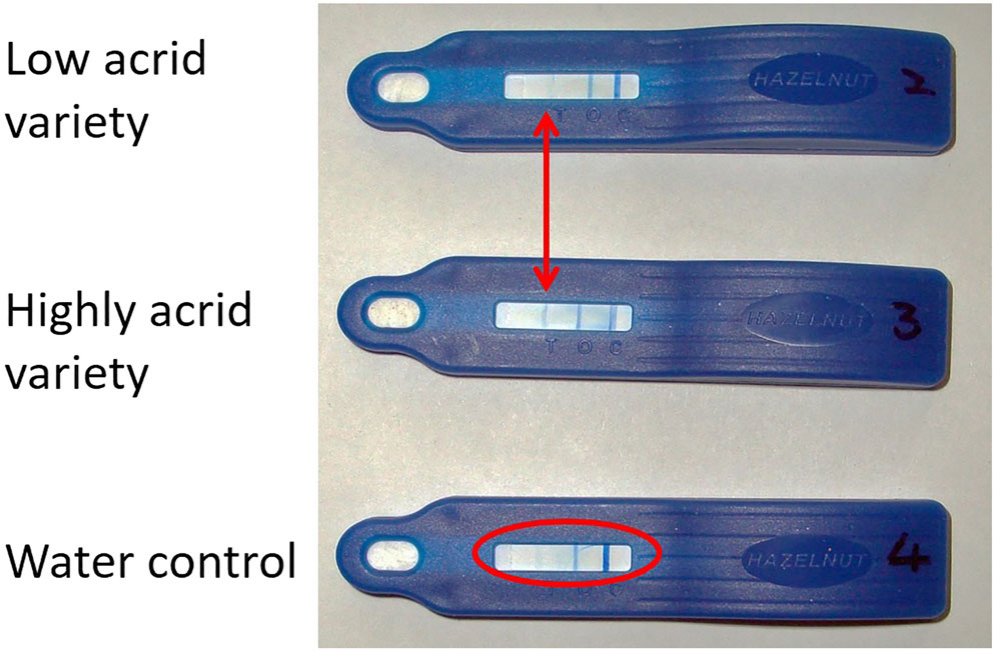
Dip test results to taro leaf extract on Neogen Hazelnut ELISA test strip. The variety with low acridity was compared with a variety with high acridity. The low acrid and water control show only two second control bands, while the highly acrid variety (99‐6) showed a faint third band that was consistently seen in a number of replications. *n* = 3.

Two taro profilins (EVM0004272 and EVM0017269) had amino acid numbers similar to our isolated cDNA clones (supporting information Table S3) and were in the expected size range (100 to 131 amino acids), while another profilin had two additional amino acids (EVM0021119), and a fourth had an insert of 17 amino acids, five of which were prolines, including a triplet (EVM0003866) (Table 2 and supporting information Table S6). There was some amino acid sequence variation across different taro profilins and between profilins of taro and other species (Figure 4 and supporting information Table S5), though all were predicted to fold into a similar structure (Davey & Moens, 2020; Jespersen et al., 2017; Krishnan & Moens, 2009). The two taro profilins with the inserts had a higher pI (6.05 and 6.4 versus 4.51 and 4.73) and had fewer positively and negatively charged amino acids than the other raphide‐associated proteins. All the taro profilins showed consistently four B‐cell epitopes above the default threshold value of .5 (Fleri et al., 2017; Jespersen et al., 2017). The profilin with the larger insert was the most highly expressed profilin found on the raphides and had a greatly enhanced B‐cell epitope prediction of .724 (35% increase) in the second B‐cell epitope (Figure 5) that was an exposed coiled region (supporting information Table S6). This exposed epitope provides a site for greater IgE antibody binding and potential allergenicity (Pomés, 2010), though it does not predict immunogenicity and its clinical manifestation (Paul, 2012; Radauer et al., 2006; Sanchez‐Trincado et al., 2017). Our result shows that different profilins can potentially induce a range of different immunogenic responses and explains the differences in acridity among different aroids and taro cultivars. The cross‐reactivity with commercial polysomal lateral flow hazelnut tests (Figure 6) where profilins are regarded as a secondary antigen opens the possibility for a similar device for screening acridity.

### 4.3 Potential mechanism for profilin allergenicity

Profilins affect cell shape and function via actin filament formation (Davey & Moens, 2020; Pandey & Chaudhary, 2020). Profilin can inhibit actin polymerization into F‐actin by binding to monomeric actin, but profilin may also promote actin polymerization in absence of F‐actin capping protein. The presences on the raphides of both actin and profilins (Figure 1a,b) suggest a potential mechanism for raphide biomineralization with F‐actin acting as a guide for the crystallization of calcium oxalate as raphides. The synthesis of these raphide crystals is regarded as a complex, coordinated process (Arnott & Webb, 2000; Kausch & Horner, 1984; Pennisi et al., 2001) that occurs in a rounded membrane chamber (Arnott & Pautard, 1970; Horner & Whitmoyer, 1972; Pennisi et al., 2001; Webb, 1999; Webb et al., 1995). Crystal growth (biomineralization) occurs in this vacuolar, membranous chamber in which the calcium oxalate crystallizes (Arnott & Pautard, 1970; Horner & Wagner, 1995; Kostman et al., 2001; Li et al., 2014; Webb, 1999; Weiner & Addadi, 2011). The genes for the synthesis of oxalic acid potentially derived from ascorbic acid (Kostman et al., 2001) are expressed in taro apex (Figure 2b) with several calcium channels and exchangers (Figure 2a). The coordination of these processes during early raphide development could explain calcium being incorporated along the whole length of the raphide (Kostman et al., 2001; Li et al., 2014), then only at the ends during the raphide's maturing phase (Franceschi, 1989). A number of polypeptides are associated with the calcium oxalate crystal matrices of grape, tobacco, taro, tomato, and water lettuce (Bouropoulos et al., 2001; Li et al., 2003, 2014; Paull et al., 1999; Webb, 1999; Webb et al., 1995). The proteins associated with raphide biosynthesis within the idioblast would be expected to remain closely associated with the mature raphide surface.

The solubility product (K_sp_) of calcium oxalate monohydrate at physiological temperatures (25°C) and pH (6 to 7.55) is 6.7 × 10^−9^ (mol^2^/L^2^), about twofold lower than in pure water, with pH having the greatest effect (Ibis et al., 2020). The typical pH of the cytosol is ~7.2 versus 5.2 in the vacuole, with the trans‐Golgi network and multivesicular bodies being about 6.3 (Shen et al., 2013). The controlled release of calcium from the vacuole into the narrowly confined “crystal chamber” with limited free water and a supply of oxalate from associated mitochondria and peroxisomes would soon exceed the calcium oxalate K_sp_ (supersaturation) and lead to calcium oxalate nucleation and crystallization. Though not studied in plants, this supersaturation and crystallization has been studied in urine where oxalate concentration above .15 mM leads to crystal formation (Berland et al., 1988). Development of the needle‐like raphide morphology in the idioblast and prevention of fusion of raphides developing in bundles suggests possible roles for calcium‐binding proteins and self‐assembled organic template that may be proteins (Li et al., 2014; Webb, 1999). Demineralization of immature *Yucca brevifolia* raphides found an organic structure that was not characterized. In a paper on grape raphides (Webb et al., 1995), the organic crystal matrix was found to promote formation of raphide‐like crystals. Consistent with this finding (Bouropoulos et al., 2001; Li et al., 2003; Li et al., 2014), extracted macromolecules from crystals of tomato, tobacco, water lettuce, and banana promote calcium oxalate nucleation and crystal growth in vitro, leading to the proposal that one or more components of the organic matrix, possibly a protein, form a template for such precipitation and control growth morphology that leads to the needle‐like raphides occurring in bundles. Multiple matrix proteins have been partially identified in plants (Klanrit, 2006; Li et al., 2003, 2014; Webb et al., 2001). Li et al. (2014) described a protein that preferentially nucleates minerals and controls their growth morphology, for which limited sequence data suggested an unknown peptide with chaperone‐like character. A similar chaperone peptide has been found by others (Klanrit, 2006; Li et al., 2003; Webb et al., 2001), and it was later found that this protein contains a calcium‐binding domain. Neither the amino acid composition of the raphide matrix protein from water lettuce nor its molecular weight (Li et al., 2003) matches the taro raphide peptides amino acid composition from our genome analysis (supporting information Table S2). A proposed role is that these template proteins act as fibers embedded in the individual raphides that bind Ca and create a supersaturation at the peptide fiber–solution interface that leads to calcium oxalate exceeding its K_sp_ and forming a crystal (Li et al., 2014). This proposal is supported by the finding in Lemna raphides (Weber et al., 2016) that the tips were initially amorphous then crystallized, as has been reported after nucleation in in vitro systems (Hajir et al., 2014; Ihli et al., 2015) and kidney stones (Rez, 2017). We did find on the taro raphides a chaperonin (EVM0000630) with a plastid target. Though highly expressed in our apex transcriptome, this chaperonin is not predicted to bind calcium (Trösch et al., 2015; Zhao & Liu, 2018). There was limited homology between the peptide sequence in Li et al. (2014) and any taro gene observed in the genome or expressed in the apex. If the raphide template fiber was buried as a template in the developing raphide, it is unlikely that our 2D gel electrophoresis rehydration buffer would etch or demineralize the raphides and extract the proposed matrix fiber peptides. The peptides extracted by the rehydration buffer from taro raphides would then be expected to be on the surface of the raphides.

### 4.4 Potential cellular structure of raphide interactions

**FIGURE 7:**
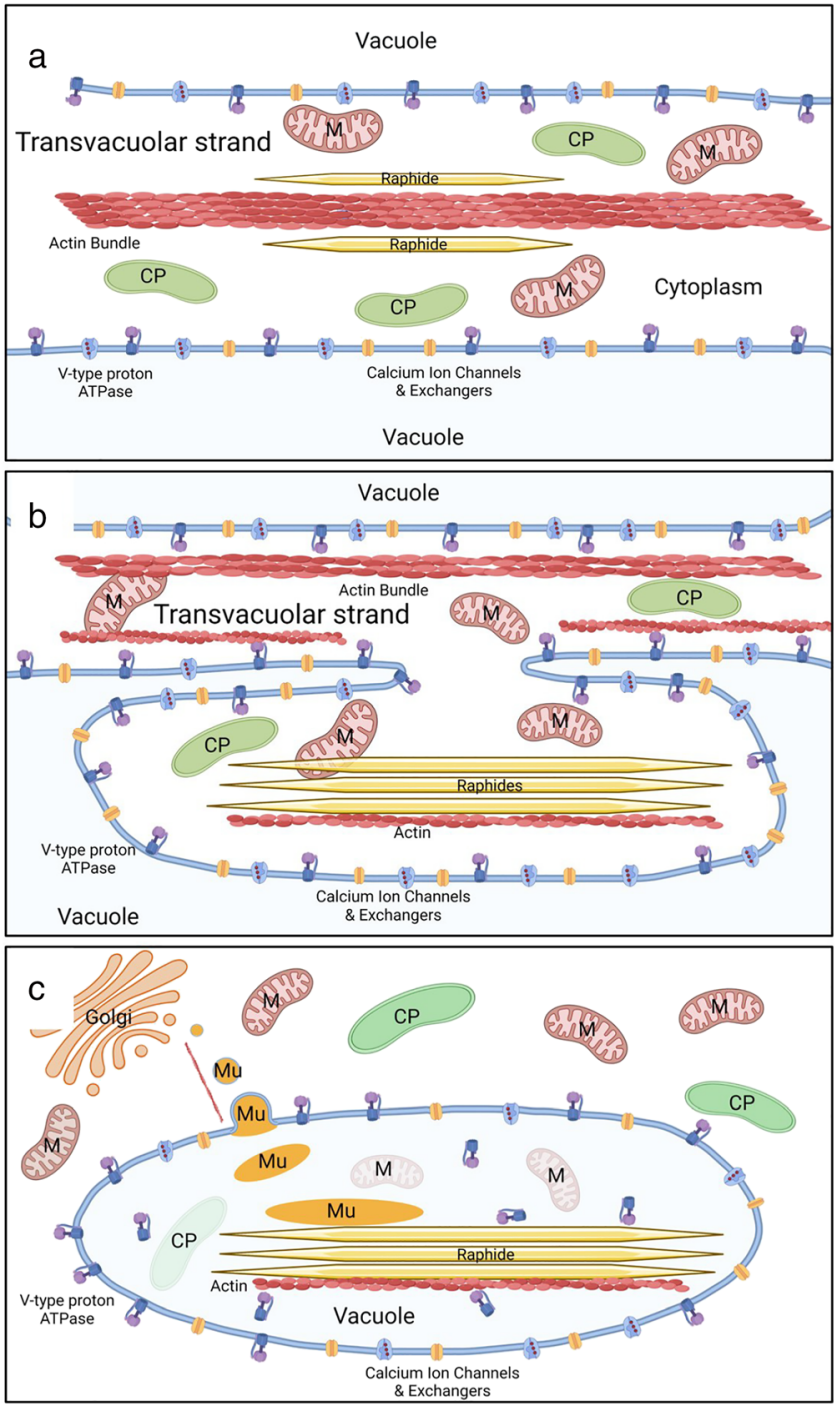
Simplified model for raphide bundle synthesis in taro, based on the peptides found associated with purified raphides and transcription data presented and the changes in organelles and crystals at different stages of cattail (*Typha augustifolia* L.) raphide development (Kausch & Horner, 1983) and other references cited in the discussion. (a) Earliest stage where actin serves as a foundation for crystal biomineralization and growth though not necessarily the initial nucleation site, potentially in transvacuolar strands or the interface between vacuoles with mitochrondria (M) and crystalloplastids (CP) closely associated with the growing raphides; (b) Intermediate stage where an invagination of the vacuolar membrane that envelopes growing raphides and actin filament bundles with some mitochondria and crystalloplastids. Endoplasmic membranes may also be engulfed in this process. A microautophagy model is indicated in this figure; macroautophagy involving the formation of a phagosome is possible. (c) Final stages with completion of raphide growth where captured mitochrondria and crystalloplastids undergo lysis and some of the peptides released from these organelles being deposited on the raphides along with mucilage synthesis (MU) transferred in vesicles from the Golgi, with actin acting as a guide. Individual figure not to the same scale. Created with BioRender.com

The complexity of the mix of peptides detected with the raphides (Table 1) and their predicted organellar targeting to vacuole, mitochondria, and crystalloplastids (dedifferentiation amyloplasts) (Kausch & Horner, 1983) (Table 2) could reflect the peptides' intimate association with the raphide or could be due to contamination during raphide purification from frozen tissue in petroleum ether. The presence of a mucilage around the raphides and the consistently narrow range of peptides found on the raphides (Table 1 and supporting information Table S1) would argue against contamination, as a similar range of peptides was found in both leaf petiole and corm. Starch is a common contaminant in raphide purification because the densities of starch and raphides are similar, but none of the raphide‐associated peptides is connected to starch granule metabolism (Buleon et al., 1998; Malinova et al., 2018). Similarly, with the exception of V‐type ATPases, common vacuolar proteins were not found (Carter et al., 2004; Jaquinod et al., 2007). Since raphide synthesis occurs soon after cell division at the apex before a central vacuole has formed, and since actin is intimately associated with organellar and vacuolar biogenesis and transport (Krüger & Schumacher, 2018; Sheahan et al., 2007; Wang et al., 2017), any biomineralization and antigen deposition model needs to include all the peptides found on the raphides. The network analysis of gene expression (Figure 3 and supporting information Table S4) in the apex supports a clustering of several genes whose products were found on the raphides, as well as genes that are involved in actin cytoskeleton, oxalate synthesis, calcium‐binding and transport, and vacuolar sorting (Cui et al., 2020; Demidchik et al., 2018; Wang et al., 2017; Zhang et al., 2014). Plant actin bundles are closely associated with transvacuolar strands and function together with myosin proteins in the strands' formation, rearrangement, and movement (Higaki et al., 2006; Sheahan et al., 2007; Wang et al., 2017; Zhang et al., 2014). Profilins occur outside of the network central clusters either alone or with an actin‐related protein (EVM0003714, 2/3 complex subunit 3) but not with actin. The changes in organelles and crystals at different stages of raphide development (Franceschi, 1989; Franceschi & Nakata, 2005; Horner & Wagner, 1995; Horner & Whitmoyer, 1972; Kausch & Horner, 1983; Kausch & Horner, 1984; Kawasaki et al., 2004; Li et al., 2014; Nakata, 2012; Prychid et al., 2008; Raman et al., 2014; Webb, 1999; Webb et al., 1995) were used to develop a simplified model (Figure 7). It is proposed that at the earliest stage, F‐actin serves as a foundation for crystal biomineralization and growth direction, though not necessarily as the initial calcium oxalate nucleation site, or as a raphide growth factor template lacking a calcium‐binding domain. F‐actin has been implicated in biomineralization and chamber morphology in foraminifera and algae (Durak et al., 2017; Tyszka et al., 2019). Profilins were potentially involved in this actin polymerization (Paez‐Garcia et al., 2018). This guided crystal growth possibly occurred in the narrow confines of transvacuolar strands or at an interface between vacuoles, with mitochrondria and crystalloplastids closely associated with the growing raphides. Vacuolar and endoplasmic reticulum biogenesis has been shown to form a very dynamic organelle that interacts with the cytoskeleton and undergoes changes in morphology through fragmentation and fusion (Breuer et al., 2017; Cao & Brandizzi, 2019; Cui et al., 2019, 2020; Kriechbaumer & Brandizzi, 2020; Krüger & Schumacher, 2018; Marty, 1999; Shimada et al., 2018; Wang et al., 1994; Zirkle, 1937). After this initial phase when raphide growth has been substantial, an invagination of the vacuolar membrane occurs that envelopes the growing raphides and actin filament bundles with some mitochondria and crystalloplastids. The engulfing could be similar to “bulb” membrane formation in vacuolar membrane (Chanoca et al., 2015; Han et al., 2015; Saito et al., 2011 & 2011). In this invagination and fusion, the V‐type proton ATPase outer B‐subunit is facing towards the raphides outside of a vacuolar vesicle. The B subunit of vacuolar H^+^‐ATPase has been shown to possess actin‐binding sites that lead F‐actin to bundle and that stabilize actin filaments (Ma et al., 2012; Wang et al., 2017; Wang et al., 2021). This actin binding could lead to the V‐ATPase being attached to the outside of the raphides. Endoplasmic membranes may also be engulfed in this process. Microautophagy that can involve tonoplast invagination was included in this model to account for proteins normally associated with mitochondria and crystalloplastids being on the raphides (Broda et al., 2018; Li & Vierstra, 2012; Liu & Bassham, 2012; Nakamura et al., 2021; Sienko et al., 2020; Stefaniak et al., 2020; Zhuang & Jiang, 2019). Macroautophagy involving the formation of a phagosome was not excluded from this model. In the final stage, raphide growth ceases, engulfed, and captured mitochrondria, and crystalloplastids undergo lysis (Kawasaki et al., 2004); some of the peptides released from these organelles are deposited around the raphides, along with mucilage (Kausch & Horner, 1983; Sakai et al., 1972; Wang et al., 1994) that is synthesized and transferred to the raphides in vesicles, likely from the Golgi, with actin filaments acting as a guide (Kim et al., 2020).

### 4.5 Implications for raphide evolution

The wide distribution of needle‐shaped raphides (Franceschi & Horner, 1980; Franceschi & Nakata, 2005; Prychid & Rudall, 1999; Raman et al., 2014; Tang & Sakai, 1983) suggests a common ancestor. It has been shown in a crystal nonaccumulating plant (Arabidopsis) that some of the underlying crystal‐forming machinery is conserved (Nakata, 2012, 2015), and this might have utility in plant disease control. Gilbert et al. (2022) reviewed biomineralization of calcium carbonate and concluded that it evolved independently and convergently across all phyla. The evolution of needle‐shaped raphides in plants may serve an antifeeding deterrent in cases such as described by Stahl (1888) for snails and the weak response with silkworms (Konno et al., 2014). In other species, the addition of an allergen may enhance the raphides' antiherbivory potential, though how this plant defense response is coupled to plant resource allocation and fitness is unclear (Hanley et al., 2007). Konno et al. (2014) showed a synergism between kiwi fruit raphides and a cysteine protease. Our results indicate that profilin was a likely allergen on the surface of raphides that may serve as carriers for the underlying cause of acridity. This conclusion would explain the variation in sensitivity of individuals to taro acridity and the absence of acridity in many raphide‐containing plants (Tang & Sakai, 1983). However, this hypothesis as to the role of profilins maybe be limited to Colocasia spp., with Dieffenbachia acridity potentially being due to a protease as reported by ourselves (Paull et al., 1999) and others (Fochtman et al., 1969; Walter & Khanna, 1972). The presence of multiple peptides on taro raphides provides some insights into raphide formation, though not into the initial stages of crystal nucleation and morphological development, with our data suggesting a role for actin being involved either in linear crystal growth or in the guidance of organelles to the raphide bundles.

## CONFLICT OF INTEREST

The authors declare no conflict of interest.

## AUTHOR CONTRIBUTIONS

R.E.P conceptualized the research, had overall supervision, and administered of the project; R.E.P., N.J.C., D.Z‐C., and C.M.J.W were involved in data curation; R.E.P., N.J.C., G.U., M.K., D.Z‐C., and C.M.J.W. performed the formal analysis and validation; R.E.P. acquired the funding and secured the resources; R.E.P., N.J.C., G.U., D.Z‐C., C.M.J.W., and M.K. conducted the research and investigation process; R.E.P. G.U., and M.K. contributed the analytical tools and analyzed the data and created the model and graphs; R.E.P wrote the original draft of the manuscript, and R.E.P., D.Z‐C., and M.K. were involved in the review and editing of this manuscript. The final manuscript was approved by all the authors. The author(s) responsible for distribution of materials integral to the findings presented in this article is Robert E. Paull (paull@hawaii.edu).

## Supporting information


**Figure S1.** Two‐dimensional gel separation of proteins from purified raphides extracted from leaf petioles of A. a low‐acridity cultivar ‘Bin Liang’, also called Chinese taro, and B. a highly acrid breeding line, 99–6.
**Table S1.** Raphide peptide mass sequencing to genome.
**Table S2.** Raphide peptide amino acid compliment.
**Table S3.** cDNA clone homology.
**Table S4.** Network cluster coefficients.
**Table S5.** Taro profilin sequence alignment to other plant profilins.
**Table S6.** Taro profilin structural and B‐cell epitope analysis.
**Table S7.** Cytoskeletal genes expressed in taro apex.
**Table S8.** Vacuolar transport, sorting and associated genes expressed in taro apex.

## Data Availability

Sequence from this study can be downloaded from NCBI GEO Website under accession GSE191235 (https://www.ncbi.nlm.nih.gov/geo/query/acc.cgi?acc=GSE191235).
